# Neuroprotective effects of carvacrol against Alzheimer’s disease and other neurodegenerative diseases: A review

**DOI:** 10.22038/AJP.2022.19491

**Published:** 2022

**Authors:** Zahra Azizi, Nahid Majlessi, Samira Choopani, Nasser Naghdi

**Affiliations:** *Department of Physiology and Pharmacology, Pasteur Institute of Iran, Tehran, Iran*

**Keywords:** Carvacrol, Alzheimer’s disease, Neurodegenerative Neuroprotection

## Abstract

**Objective:**

Neurodegenerative diseases are considered an important cause of cognitive deficit and morbidity in old ages. Alzheimer’s disease (AD) is one of these disorders affecting about 40 million people in the world at the present time. Available drug therapy is mostly symptomatic and does not modify or stop disease progression. Recently, biologically active chemicals from herbs have been studied to develop new therapeutic drugs. Carvacrol has shown positive properties on many neurological diseases. This compound is expected to have the ability to affect AD pathogenesis and therefore, it is considered an anti-AD agent.

**Materials and Methods:**

This review was conducted using PubMed, Google Scholar and Science Direct bibliographic databases until November 2021. For data collection, the following keywords were used: carvacrol, neuroprotective, cognition, anti-inflammatory, antioxidant, Acetylcolinesterase inhibitor (AChEI), Alzheimer's, Parkinson’s, epilepsy, stroke, ischemic brain injury, and neurodegenerative diseases.

**Results:**

This review summarizes *in vitro* and *in vivo* studies on protective potential of carvacrol in neurodegenerative disorders and various underlying mechanisms, such as anti-inflammatory, antioxidant, and anticholinesterase effects.

**Conclusion:**

We gave an overview of available literature concerning neuroprotective effects of carvacrol in ameliorating the neurodegenerative diseases symptoms *in vivo* and *in vitro*. Particular attention is given to AD. Several neuro-pharmacological actions of carvacrol have been summarized in the current review article including anti-inflammatory, antioxidant, and AChEI properties.

## Introduction

Neurodegenerative diseases are a heterogeneous group of disorders characterized by progressive deterioration 

of structure and functionality of the nervous system. Alzheimer's disease (AD) can be considered a main age-related dementia in the world, showing increasing incidence as a consequence of the population aging. Multiple factors play important roles in the disease pathophysiology including amyloid beta (Aβ) plaques, tau tangles, oxidative stress, and inflammation and decreased levels of acetylcholine (Carreiras et al., 2013 ▶; Eratne et al., 2018 ▶).

 The U.S. Food and Drug Administration (FDA) has approved rivastigmine, galantamine, donepezil and memantine for the treatment of AD, but none of these drugs are specifically approved to treat behavioral and psychiatric symptoms in the moderate and severe stages of AD (Weller and Budson, 2018; Mimica and Presecki, 2009 ▶). Recently, several natural plant compounds have been investigated for their potential ability to affect AD pathogenesis or reduce the disease symptoms (Anekondra and Reddy, 2005 ▶; Ansari and Khodagholi, 2013 ▶). 

 Carvacrol (5-isopropyl-2-methylphenol) a natural monoterpenoid phenol is one of the major constituents in the essential oil of several aromatic plant including *Thymus, Origanum*, *Zataria*, *Thymbra*, *Satureja*, and *Coridothymus* of the Lamiaceae and *Lippia* of Verbenaceae family (Baser, 2008 ▶). Recently, increasing attention has been given to define pharmacological effects of carvacrol. In last years, carvacrol obtained FDA approval as a food flavoring ingredient and preservative, that could be added in low concentrations to some food like baked goods, and chewing gum (Silva et al., 2015 ▶; De Vincenzi et al., 2004 ▶). In addition, carvacrol has been widely utilized as a cosmetic essence (Zotti et al., 2013 ▶). So far, toxicological data has shown no toxicity for carvacrol (Silva et al., 2015 ▶; De Vincenzi et al., 2004 ▶). 

 Carvacrol is able to cross the blood brain barrier easily, notably improving its therapeutic efficacy in neurodegenerative disorders (Lee et al., 2020 ▶). A variety of methodological designs in different animal and cellular models has shown diverse bioactivities for carvacrol such as antimicrobial (Kachur and Suntres, 2020 ▶; de Oliveira et al., 2020 ▶), antifungal (Zhang et al., 2019 ▶; Kim et al., 2019 ▶), antiviral (Mediouni et al., 2020 ▶; Gilling et al., 2014 ▶), insecticidal (Park et al., 2017 ▶; Baser, 2008 ▶), anticarcinogenic properties (Rojas-Armas, 2020 ▶; Sivaranjani, 2016 ▶; Karkabounas, 2006 ▶), anti-obesity (Suganthi and Manpal, 2013 ▶), antioxidant (sharifi-Rad et al., 2018 ▶; Tiefensee Ribeiro et al., 2019 ▶; Naeem et al., 2021 ▶; Delgado-Marín et al., 2017 ▶), analgesic (Horishita et al., 2020 ▶), anesthetic (Bianchini et al., 2017 ▶), antispasmodic (Boskabady et al., 2012 ▶; Baser, 2008 ▶), anti-inflammatory (Li et al., 2016 ▶; Baser, 2008 ▶), Acetylcolinesterase (AChE) inhibitory (Kurt et al., 2020 ▶; Kaufmann et al., 2011 ▶; de Souza et al., 2020 ▶; Baser, 2008 ▶) and anti-hepatotoxic activities (Khan et al., 2019 ▶). These pharmacological activities have potential role to the treatment and prevention of many chronic diseases, such as cancer as well as infectious, cardiovascular and neurodegenerative diseases (Silva et al., 2015 ▶; Friedman, 2014 ▶). 

 In vivo studies have presented diverse pharmacological effects of carvacrol including antioxidant, anti-inflammatory, and memory-enhancing activities (Sharifi-Rad et al., 2018 ▶; Salehi, 2018). A variety of animal models such as AD (Azizi, et al., 2012 ▶; Asadbegi et al. 2017; Guan et al., 2019 ▶), Parkinson’s disease (PD) (Manouchehrabadi et al., 2020 ▶; Lins et al., 2018 ▶; Dati et al., 2017 ▶), cerebral ischemia (Hong et al, 2018 ▶; Chen et al., 2015 ▶; Yu et al., 2012 ▶) and stroke (Hong et al., 2018 ▶; Li et al., 2016 ▶; Chen et al., 2015 ▶) has reported neuroprotective abilities of this compound. 

Neurodegenerative diseases are considered an important cause of cognitive deficit and morbidity in old ages. Extensive researches have revealed carvacrol neuroprotective properties that could be useful in preventing or ameliorating neurodegenerative diseases *in vitro* and *in vivo*. This article comprehensively reviews the mechanisms of action of carvacrol including antioxidant, anti-inflammatory, and AChE inhibitory effects in *in vivo* and *in vitro* AD models as well as several of the most common neurodegenerative diseases particularly those associated with cognitive impairment, hoping to provide some ideas for identiﬁcation of carvacrol neuroprotection.

## Materials and Methods

This review was conducted using PubMed, Google Scholar and Science Direct bibliographic databases until November 2021. For data collection, the following keywords were used: carvacrol, neuroprotective, cognition, anti-inflammatory, antioxidant, acetylcholinesterase inhibitor, Alzheimer's, Parkinson’s, epilepsy, stroke, ischemic brain injury, and neurodegenerative diseases. The search strategy design was including all available literature evaluating carvacrol with at least one of the above keywords. In most cases, the original full-text articles were obtained and appropriate data was extracted. The search was limited to English language. 

## Results

**Carvacrol mechanism of action in preventing of neurodegenerative diseases**

**Antioxidant activity**

High levels of reactive oxygen species (ROS) levels induce oxidative stress that was revealed to contribute to cell death and neurodegeneration progression (Swomley and Butterfield, 2015 ▶). Oxidative stress has been reported as an underlying cause of many diseases like PD, AD, cardiovascular disease, and diabetes. Therefore, therapeutic agents able to attenuate oxidative stress might be beneficial in AD treatment (Aliev et al., 2008 ▶). Carvacrol has antioxidant activity and was shown to act as a dietary phyto-additive to boost animal antioxidant status (sharifi-Rad et al., 2018 ▶). Moreover, carvacrol could protect neuronal injuries against Aluminum-induced oxidative stress leading to lipid peroxidation (Baranauskaite et al., 2020 ▶). It was shown that carvacrol could be able to scavenge intracellular ROS and exert neuroprotective effects against oxidative stress induced apoptosis (Zamanian et al., 2021 ▶). Carvacrol was shown to inhibit 3-nitrotyrosin and malondialdehyde formation. These outcomes specifically support carvacrol effect to prevent toxic elements formation by reactive nitrogen species action (Prieto et al., 2007 ▶). Moreover, carvacrol has been indicated to reduce malondialdehyde (MDA) and neuronal cell necrosis, and increase superoxide dismutase (SOD) and catalase (CAT) activity levels in the hippocampus (Shahrokhi Raeini et al., 2020 ▶). In addition, carvacrol activated nuclear factor-erythroid 2-related factor 2 (Nrf2) as an endogenous antioxidant, which could further regulate downstream antioxidants expression (Naeem et al., 2021 ▶). Moreover, carvacrol modulated B-cell lymphoma 2 (Bcl-2) protein expression, Bcl2-associated X protein (Bax), caspase-3, and phosphorylated Extracellular Signal-Regulated Kinase (p-ERK)(Wang et al., 2017 ▶). Another study indicated that carvacrol co-exposed with cadmium elevated glutathione cellular levels and increased glutathione reductase expression in PC12 cells. The cadmium-induced down-regulations of Nrf2, extracellular signal-regulated kinase-1 (ERK-1), protein kinase B (Akt), and nuclear factor kappa-light-chain-enhancer of activated B cells (NFКB) expressions were reversed by carvacrol (Banik et al., 2019 ▶). 

**Anti-inflammatory activity**

Recently, inflammation was identified as a main underlying cause of neurodegenerative diseases such as AD, PD, ischemic stroke and epilepsy, making neuroinflammation a crucial therapeutic target in neuronal disorders (Lee et al., 2020 ▶). Neuroinflammation is linked to Aβ plaque and neurofibrillary tangle formation and plays a significant function in the pathophysiology of AD (Zhang and Jiang, 2015 ▶). The progressive cognitive impairment has been correlated with increased levels of inflammatory markers. Thus, novel therapeutic process intended at these pathophysiological components of AD are purposefully developed now (Vaiserman et al., 2020 ▶). 

Carvacrol anti-inflammatory effect was verified in rats and in several cellular models of inflammation (Tiefensee Ribeiro et al., 2019 ▶). This effect was investigated in lipopolysaccharide-induced-neuroinflammation and memory impairment in a rat model (Lee et al., 2020 ▶). Carvacrol administration (25, 50, and 100 mg/kg) during 21 days attenuated memory impairments and enhanced cognition compared to the control group. Carvacrol administration diminished the expression of interleukin-1β (IL-1β), cyclooxygenase-2 (COX-2), and tumor necrosis factor-α (TNF-α). Moreover, carvacrol could significantly decrease Toll-like receptor 4 (TLR4) and increase brain-derived neurotrophic factor (BDNF) expression. This cognitive increase comes from the anti-inflammatory effect of carvacrol mediated by BDNF and TLR4 regulation. Thus, carvacrol exhibited a promising potential to inhibit memory impairment in neurodegenerative disorders (Lee et al., 2020 ▶). Treatment with carvacrol prevented the cytokines levels, the activity of myeloperoxidase (MPO), and COX-2 and inducible nitric oxide synthase (iNOS) expression. Carvacrol also decreased MDA level and induced SOD activity in ischemic cortical tissues (Li et al., 2016 ▶). 

Moreover, SH-SY5Y cells exposure to Fe(2+) diminished the cell viability, that was reduced by carvacrol pretreatment. Nuclear factor kappa B (NF-κB) activation, cell apoptosis and expression of pro-inflammatory cytokines induced by Fe(2+) were significantly inhibited by carvacrol. Moreover, carvacrol suppressed c-Jun N-terminal kinase (JNK) and IκB kinase (IKK) phosphorylation induced by Fe(2+) (Cui et al., 2015 ▶).

Additionally, the neuroprotective ability of carvacrol on neuronal inflammation, oxidative stress, and learning and memory in lipopolysaccharide (LPS)-treated rats were assessed. MDA, IL-6 and nitric oxide (NO) metabolites were elevated in the brain by LPS injection, while CAT, thiol and SOD were decreased. Carvacrol pretreatment could reduce MDA, IL-6 and NO, while increased CAT, thiol and SOD (Hakimi et al., 2020 ▶). In another research, Celik et al. have shown that carvacrol decreased the pro-inflammatory response to methotrexate that causes inflammation and oxidative stress in sciatic nerve tissues of rats (Celik et al., 2013 ▶). Carvacrol also prevented pro-inflammatory effects through down regulation of NF-κB transcription in SH-SY5Y cells treated with H_2_O_2_. Thus, it was suggested that carvacrol has cytoprotective effects in SH-SY5Y cell line following H_2_O_2_ exposure (Chenet et al., 2019 ▶).

**Acetylcholinesterase inhibitory effects**

Cholinergic system is mostly disrupted in AD, which results in acetylcholine (ACh) level decrease in the brain area managing learning and memory. The common goal of drug therapy in AD is to prevent ACh level reduction (Liu et al., 2017 ▶; Wang et al., 2017 ▶). While inhibitors of AChE provide beneficial effects in AD, their efficacy to stop disease progression is low (León et al., 2013 ▶). Therefore, AChEI activity of phytochemicals is interesting as a possible treatment of AD. Numerous researches indicated that some components of plant essential oils such as carvacrol, have the ability to inhibit cholinesterase activity (Burčul et al., 2020 ▶). Preliminary studies revealed that carvacrol has AChEI activity (Orhan et al., 2008 ▶; Jukic et al., 2007 ▶). In another study, selected constituents of essential oils including carvacrol were examined for their *in-vitro* AChEI activity and the results showed that carvacrol apparently inhibited AChE (Kaufmann et al., 2011 ▶). As the present therapeutic strategy in AD patients is to restore function of cholinergic system via inhibiting AChE and consequently enhancing cholinergic neurotransmission, the activity of carvacrol in AChE inhibition appears to play a valuable role in cognitive function improvement (Azizi et al., 2012 ▶).

**Neuroprotective effect of carvacrol in neurodegenerative diseases**

Neurodegenerative diseases are characterized by progressive structural abnormalities and dysfunction of the central nervous system (CNS), typically occurring in elderly people. Unfortunately, there is no treatment for preventing or slowing the progression and development of such diseases. Thus, investigation for novel strategy and therapeutic agents has become an important topic for research (Wang et al., 2020 ▶).

Antioxidative properties of carvacrol are related to the phenolic OH group, and the hydrophobic properties are associated to the benzene ring and the isopropyl and methyl substituents. These properties appear to contribute to the modulation of several neurodegenerative diseases (Friedman, 2014 ▶). 

**Anti-Alzheimer’s disease activity**

Alzheimer’s disease is one of the main causes of dementia that affects cognitive function (Duyckaerts et al., 2009 ▶). Several plants and phytochemical compounds exhibit antioxidant, anti-inflammatory and AChE inhibitory capacities and therefore, can be valuable in the management of neurodegenerative diseases especially cognitive disturbances related to these diseases. In our previous finding, we indicated that carvacrol has therapeutic ability in modulating or preventing AD (Azizi et al., 2020 ▶).

First, the effect of *Zataria multiflora *Boiss. (ZM) essential oil was evaluated in an AD rat model. Aβ_25-35_ was infused in the rat hippocampus CA1 region and the effect essential oil of ZM on cognition was assessed in the Morris water maze. The results showed that injection of Aβ_25-35 _induced spatial memory impairment in the rat hippocampus; however, ZM essential oil reversed this impairment. Hence, ZM or its main constituent carvacrol could be a potential valuable natural therapeutic agent for AD treatment (Majlessi et al., 2012 ▶). Later, our study demonstrated that thymol and carvacrol could be able to protect against the toxicity of Aβ_25-35_ injection or scopolamine on escape latency and traveled distance in two AD rat models and suggested the effectiveness of carvacrol and thymol in alleviating cognitive impairment (Azizi et al., 2012 ▶). An *in vitro* study in our lab showed that carvacrol and thymol could protect against Aβ_25-35_-induced PC12 cell death through protein kinase C (PKC) and ROS pathways. This study suggested that carvacrol neuroprotective effect against Aβ might be attributed to oxidative damage attenuation and activation of PKC as a protein related to memory function. Therefore, carvacrol presented therapeutic potential to prevent or modulate AD (Azizi et al., 2020 ▶). 

It has been indicated that PKCα activity could increase α-secretase activation that suppresses the production of Aβ (Murakami et. al., 2020). Furthermore, PKC activity and PKCα protein expression levels were examined in hippocampal cells from an AD rat model. We noticed that carvacrol and thymol signiﬁcantly elevated PKCα expression level in comparison with Aβ group in AD rat model. The results explained that carvacrol and thymol might have protective ability on cognitive function in AD by activation of PKC pathway (Azizi et al., 2021 ▶). 

In another research, carvacrol was determined as the main constituent of essential oils of *Origanum vulgare* and* Thymbra capitata *by GC/MS. Carvacrol inhibited AChE and lipoxygenase activity that supports its anti-inflammation and anti-Alzheimer effects (Carrasco et al., 2016 ▶). Chemical analysis of *Lavandula pubescens *Decne essential oil (LP EO) constituents uncovered 25 components, among which, carvacrol was identified as the main molecule (65.27%). EO showed significant antioxidant, AChEI, and anti-butyrylcholinesterase effects. The strong antioxidative effect and enzyme inhibition of EO could be associated to its high levels of monoterpenes including carvacrol, as indicated by its considerable bioactivity results. This research demonstrates that LP EO and carvacrol could be suitable for the management of AD (Ali-Shtayeh et al., 2020 ▶). In another study, carvacrol was found to prevent molecular, biochemical, and behavioral changes related to diabetes in rats, showing its ability to treat diabetes-induced cognitive deﬁcit. This cognitive deficit was related to oxidative, inﬂammatory, and apoptotic process (Deng et al, 2013 ▶). Carvacrol was also reported to ameliorate lipid peroxidation and neurodegeneration and attenuate neurotoxicity in the hippocampus of rats following exposure to lead (Mehrjerdi et al., 2020).

The effect of essential oil and nanoemulsion of carvacrol was also evaluated in an AD model induced by AlCl_3_. It was found that both forms were able to overcome oxidative stress through antioxidant and anti-inflammatory activities. However, the efficiency of nanoemulsion was noticeably higher in passing the blood brain barrier and modulating neuronal changes (Medhat et al., 2019 ▶).

Another research investigated the protective effect of carvacrol on cognitive deficit in streptozotocin-induced rat model of diabetes. Streptozotocin administration significantly increased rats escape latency in Morris water maze and increased neuroinflammation, oxidative stress and apoptotic cells in the brain of diabetic rats. Injection of carvacrol remarkably attenuated diabetes-associated cognitive deficit (Deng et al., 2013 ▶). Behavioral experiments showed that exposure to propiconazole (PCZ), an ergosterol biosynthesis inhibiting fungicide, affected motor, psychological, and cognitive function. Moreover, following PCZ exposure, glutathione peroxidase (GPx) and SOD activities in brain tissues decreased. Administration of carvacrol modulated PCZ-induced damaging effects via protective mechanisms related to neural function improvement and inhibition of oxidative stress and DNA injury (Elhady et al., 2019 ▶) (Table 1). 

**Anti-Parkinson’s disease**

Three major movement disorders are among PD symptoms. They are bradykinesia (slow movements), hypokinesia (reduced amplitude of movements), and akinesia (absent normal unconscious movements) and also tremor at rest and muscle rigidity. Loss of dopaminergic neurons is considered the main cause of motor associated symptoms (Manoochehrabadi et al., 2020 ▶; Le Heron et al. 2018 ▶). It has been demonstrated that oxidative stress plays an important role in the PD pathophysiology, and antioxidant and anti-inflammatory drugs might be able to attenuate dopaminergic neurodegeneration (Aryal et al., 2020 ▶; Mazo, 2017 ▶). Lins et al. suggested that carvacrol has a protective role in a PD rat model, and prevented reserpine-induced neurochemical and motor deficits. Therefore, carvacrol may be considered a promising novel therapy for the preventing or treating PD (Lins et al., 2018 ▶). Dati et al. examined carvacrol neuroprotective eﬀects on neurodegeneration induced by 6-hydroxydopamin (6-OHDA) intrastriatal administration in mice. They also assessed carvacrol behavioral eﬀects in that PD model using cylinder test, and evaluated caspase-3 levels by immunoblots and transient receptor potential melastatin (TRPM7), as major carvacrol targets.

**Table 1:** Anti-Alzheimer’s disease activities of carvacrol

Biological activity	Type of Study	Model	Rate of administration	Concentration	Outcomes	Reference
**Antioxidant ** **AChEI**	In vitro	-	-	0.16–0.18 μl/ml) 0.9 μl/ml	Signify a basis for developing a novel therapeutic strategy for AD	
**Antioxidant,** **AChEI,** **Anti-inflammatory**	In vivo	Rat (Aβ_25-35_ (Scopolamine)	i.p.	2 mg/kg	Reversed the learning deficits caused by Aβ or scopolamine	
**Antioxidant** **PKC activator**	In vitro	PC12 (Aβ_25-35_)	-	10, 20, and 50 μM	Protected PC12 cells against cytotoxicity Induced by Aβ 25-35	
**Antioxidant** **PKC activator**	In vivo	Rat (Aβ_25-35_)	i.p.	2 mg/kg	Protected cognitive function in AD models through PKC activity	
**Oxidative stress** **Anti-apoptosis**	In vitro	PC12 (cadmium)	-	100 μM	Reduced cadmium-triggered oxidative damage and apoptosis in PC12 cells	
**Antioxidant,** **PKC activator**	In vitro	PC12(Aβ_25-35_)	-	10, 20, 50 μM	Attenuated oxidative damage and increased the PKC activity	
**Anti-inflammatory; attenuated IL-1β, TNF-α, and COX-2; increased expression of BDNF mRNA, and decreased expression of TLR4 mRNA.**	In vivo	Rat (LPS)	Oral gavage (21 day)	25, 50, 100 mg/kg	Improving LPS induced memory deficit	
**Antioxidant activity**	In vivo	Rat (Lead)	Oral gavage (40 day)	25, 50, and 100 mg/kg	ameliorated lead induced learning and memory deficit	Mehrjerdi et al., 2020
**Antioxidant,** **Anti- inflammatory**	In vivo	Rat (LPS)	i.p.	25, 50, and 100 mg/kg	Protected from learning and memory impairment in LPS-treated rats	
**Antioxidant,** **Anti-inflammatory**	In vivo	Rat (AlCl3)	Orally	20 μl/Kg (twice a week for 30 days).	Carvacrol nanoemulsion and essential oil overturned AlCl3 induced AD.	
**Antioxidant,** **Anti-inflammatory,** **Anti-apoptotic**	In vivo	Rat (Streptozotocin) the model of diabetes	i.p.	25, 50, and 100 mg/kg (7 weeks)	Diabetes related molecular, biochemical and behavioral changes	
**Antioxidative stress, Antiapoptotic activity,** **modulates Bcl-2, Bax, caspase-3, and p-ERK protein expression**	In vivo, In vitro	Rat (ethanol) -Hippocampal neuron simulated by ethanol	liquid diets treatment (4 weeks)	25, 50, and 100 mg/kg -0.6, 0.8 mM	Attenuated the cognitive dysfunction	


AD, Alzheimer's disease; Aβ, Amyloid β; AChE protein kinase C; AlCl3, Aluminium chloride; Bax, Bcl2-associated X protein; Bcl-2, B-cell lymphoma 2; BDNF, Brain-derived neurotrophic factor; COX-2, Cyclooxygenase-2; IL-1 β, interleukin-1β; i.p., Intraperitoneal injection; LPS, lipopolysaccharide; mg/kg, milligrams per kilogram; mM, millimolar; μl/Kg, microliter per kilograms; p-ERK, Phosphorylated Extracellular Signal-Regulated Kinase; PKC, protein kinase C; TNF-α, Tumor necrosis factor α.

It was explained that 6-OHDA-induced asymmetrical use of the forelimbs significantly decreased by carvacrol. The level of caspase-3 increased after injection of 6-OHDA, while carvacrol seemed to inverse this effect. So, carvacrol was suggested to promote remarkable neuroprotection in this PD model (Dati et al., 2017 ▶). Haddadi et al. investigated carvacrol neuroprotective properties on memory and motor impairments and also hyperalgesia in a rat model of PD induced by 6-OHDA. The 6-OHDA was injected into the animals medial forebrain bundle and treatment with carvacrol was done for six weeks after surgery. The results indicated that carvacrol could significantly ameliorate memory impairments, and had no effect on rotation and hyperalgesia in lesioned rats. Carvacrol attenuated memory deficits in a PD rat model and may serve for alleviating memory impairments in PD (Haddadi et al., 2017 ▶). Another study showed that carvacrol could improve catalepsy, locomotor activity, motor coordination and bradykinesia, and reduced rotation caused by apomorphine in 6-OHDA-treated rats. Moreover, treatment with carvacrol inversed reduced glutathione level and caused a decrease in malondialdehyde (MDA) level in the 6-OHDA-treated rats (Manoochehrabadi et al., 2020 ▶). Another study of the neuroprotective properties of carvacrol and exercise on cognitive deficit in the 6-OHDA-lesioned PD rat model. Both carvacrol treatment and exercise improved memory deficit and reduced rotational behavior, which was accompanied by increased total thiol concentration and decreased lipid peroxidation in the hippocampus and striatum (Hamzehloei et al., 2019 ▶). Tiefensee Ribeiro et al. analyzed the neuroprotective effect of oral treatment with carvacrol/ β-cyclodextrin complex against 6-OHDA-induced dopamine denervation. Carvacrol and β-cyclodextrin prevented 6-OHDA-induced dopamine neuronal loss in rats. This neuroprotective activity may result from antioxidant and anti-inflammatory effects of carvacrol, as interleukin-1β (IL-1β) and Tumor necrosis factor α (TNF-α) release was inhibited by carvacrol/ β-cyclodextrin pretreatment (Tiefensee Ribeiro et al., 2019 ▶) (Table 2). 

**Table 2:** Anti-Parkinson’s disease properties of carvacrol

Biological activity	Type of Study	model	Rate of administration	Concentration	Outcomes	Reference
**Reduced caspase-3 levels, and ** **TRPM7 channels inhibitor**	In vivo	Mice (6 OHDA)	i.p.	40 mg/kg	Reduced the asymmetrical use of the forelimbs induced by 6-OHDA	
**Improved memory impairments **	In vivo	Rat (6-OHDA)	i.p.	25 mg/kg For 6 weeks after surgery	Improved memory impairments in rats with PD	
**Antioxidant activity**	In vivo	Rat (6-OHDAl)	i.p. (7 weeks)	25 mg/kg/day	Ameliorated motor and memory deficits.	
**Antioxidant activity, Increased glutathione level and decreased content of MDA **	In vitro; In vivo	PC12 cells; Rat (6-OHA)	i.p.	*In vitro*: 100, 200, and 400 μM *In vivo*: 10, 15, and 20 mg/kg/days (15 days)	Improved the locomotor activity, motor coordination, bradykinesia, and motor coordination and reduced the apomorphine-induced rotation in 6-OHDA rats.	
**Anti-inflammatory, Antioxidant**	In vivo	Rat (6-OHDA)	Oral	25 μg/kg/day (15 days)	Prevented the loss of DA neurons	
**Antioxidant**	In vivo	Rat (reserpine)	i.p.	12.5, and 25 mg/kg	Preventing neurochemical and motor deficits	


6-OHDA, 6-Hydroxydopamin; i.p., Intraperitoneal; MDA, malondialdehyde; PD, Parkinson’s disease; TRPM7, transient receptor potential melastatin

**Anti-ischemic, anti-seizure and other neurotoxicity effects**

Cerebral ischemia is one of the most common types of neuronal damage characterized by a decrease in hippocampal neurons number and their functionality. However, there is no optimal treatment for cerebral ischemia-induced neuronal injury. Review of the literature revealed a remarkable neuroprotective action of carvacrol in cerebral ischemia in animal models (Guan et al., 2019 ▶; Chen et al., 2015 ▶; Yu et al., 2012 ▶). 

Carvacrol neuroprotective effect on cerebral ischemia in mice induced by occlusion of middle cerebral artery, was evaluated. The data presented that treatment with carvacrol could significantly increase the phosphorylated protein kinase B (AKt) level, and demonstrated that the phosphatidylinositol-3-kinase (PI3K)/Akt pathway is involved in the anti-apoptotic mechanisms of carvacrol (Yu et al., 2012 ▶). In another study, carvacrol was shown to reduce cell death and inhibit ferroptosis through increasing protein expression of glutathione peroxidase 4 (GPx4). 

Moreover, carvacrol could significantly protect the gerbil hippocampal neurons via ferroptosis inhibition by elevating GPx4 expression (Guan et al., 2019 ▶). Carvacrol could also prevent hypoxic-ischemic brain damage in neonates as shown by behavioral outcome improvement, pro-survival signaling promotion, pro-apoptotic signaling inhibition and reducing brain infarct volume. Carvacrol neuroprotective effects may be mediated through inhibiting the function of TRPM7 channel (Chen et al., 2015 ▶). Hong et al. examined carvacrol neuroprotective effect on blockade of zinc influx following global cerebral ischemia (GCI). They found that the number of degenerating neurons, oxidative damage, microglial activation, and zinc translocation following GCI were significantly decreased by carvacrol administration through TRPM7 channels down regulation (Hong et al., 2018 ▶).

Another common disorder of the CNS is epilepsy. Though current anti-epileptic drug therapy could control the seizures, some patients still suffer from epilepsy resistant to medications and they are affecting by several side effects. So, a number of researches are conducted to discover new antiepileptic constituents from herbal medicines (Aghdash, 2021 ▶; Liu et al., 2017 ▶). Carvacrol could effectively inhibit recurrent status epileptics (SE) and early seizures following SE in the epilepsy perforant path model, and improve severity of disease in early stages. Carvacrol was also effective in post-SE cognitive deficit prevention in a rat model (Khalil et al., 2017 ▶). Therapeutic efficiency of carvacrol and 2-aminoethoxydiphenyl borate (2-APB) against seizure induced by pilocarpine was evaluated by Jeong et al. They found that carvacrol and 2-APB reduced the overexpression of TRPM7 induced by seizure, accumulation of zinc, and production of ROS in the cells. Additionally, apoptotic neuronal death induced by seizure was significantly decreased by carvacrol and 2-APB (Jeong et al., 2020 ▶). Carvacrol neuroprotective effect on LPS-induced seizure and possible involvement of hippocampal cyclooxygenase 1 (COX-1) and 2 (COX-2) activity were examined by Sadegh et al. Carvacrol prevented the LPS pro-convulsant effect possibly via COX-2 activity inhibition (Sadegh et al., 2018 ▶).

Chronic cerebral hypoperfusion (CCH) is considered a common experience in some neurological diseases including AD and vascular dementia (Zhao and Gong, 2015 ▶). Carvacrol presented remarkable neuroprotective properties against CCH-induced neuronal injury as it significantly attenuated learning and memory impairment tested by Morris water maze test (Shahrokhi Raeini et al., 2020 ▶)

Neuronal death caused by traumatic brain damage is a complicated process. Transient receptor potential channels inhibition has been suggested as a useful strategy to prevent CNS neuronal death. Carvacrol could inhibit transient receptor potential channel and cause a significant enhancement of traumatic brain damage recovery in mice (Peters et al., 2012 ▶). 

This compound also reduced edema and neurological deﬁcit on induced cerebral edema in a mice model, indicating its protective effect against intra-cerebral hemorrhage damage through attenuating aquaporin-4 (AQP4)-mediated edema (Zhong et al., 2013 ▶). Carvacrol also decreased the release of lactate dehydrogenase, caspase-3 activation and apoptosis following traumatic cortical neurons injury. Calcium imaging showed that cytoplasmic calcium alleviation accompanied theses effects. Moreover, neuronal nitric oxide synthase (nNOS) induction caused by traumatic injury was significantly inhibited by carvacrol. Therefore, inhibitory effect of carvacrol on TRPM7 activity might be protective against traumatic neuronal damage (Li et al., 2015 ▶).

Chronic alcohol consumption impairs hippocampal neurons and is correlated with apoptosis and oxidative stress. Wang et al. examined *in vitro* and *in vivo* neuroprotective properties of carvacrol. The results displayed that carvacrol decreases memory dysfunction and oxidative stress in the ethanol-received mice and ameliorates ethanol-induced hippocampal neurons apoptosis *in vitro*. Their results suggested that hippocampal neuron impairment mediated by ethanol was alleviated by carvacrol via antioxidant and antiapoptotic activities (Wang et al., 2017 ▶). 

Reduced amounts of neurotrophins and bioenergetics failure and abnormalities in axonal and synaptic plasticity, have been found to relate with pathogenesis of neurodegenerative diseases, such as AD and PD. Carvacrol induces neurite outgrowth by the Nerve Growth Factor (NGF) high-affinity tropomyosin receptor kinase A (trkA) activation and downstream mitogen-activated protein kinases/extracellular signal-regulated kinases (MAPK-ERK) and PI3K-AKT pathways, without depending on NGF. This study showed neurotrophic mechanisms of carvacrol that could be valuable in neurological and neurodegenerative disorders (Sisti et al., 2021 ▶).

In another study, negative behavioral effects of PCZ were investigated in rats and neuroprotective effect of carvacrol was evaluated. Behavioral assessment showed the toxic effect of PCZ on brain cognitive and motor functions. Most of PCZ adverse effects were attenuated by carvacrol treatment (Noshy et al., 2018 ▶) (Table 3). 

## Discussion

Taken together, available literature suggests that carvacrol might be a potentially valuable therapeutic agent for neuroprotection and cognitive function improvement, which may be due to its multifunctional activities including antioxidant and anti-inflammatory, and AChEI properties. Moreover, carvacrol possesses various neurotoxic effects such as anti-Parkinson’s disease, anti-ischemic, and anti-epilepsy effect. 

However, despite the variety of mechanistic studies on the neuroprotective activity of carvacrol, lack of clinical trials on the therapeutic effects of carvacrol is an important limitation that can be noted. Therefore, an accurate strategy for carvacrol administration should be designed to investigate the effect of carvacrol on early stage of Alzheimer's disease and other neurodegenerative diseases. So, further studies are needed to define its clinical efficiency, before recommending its use as a therapy for any disease.

**Table 3:** Anti-ischemic, anti-epilepsy and other neurotoxicity effects

Biological activity	Type of Study	Model	Disease	Rate of administration	Concentration	Outcomes	Reference
**TRPM7 channels inhibitor**	In vivo	Rat	status epilepticus	i.p.	75 mg/kg	Protective effects against status epilepticus -induced cell death	
**Decreased the lipid peroxide; increased the GPx4 expression**	In vivo	Gerbils(bilateral carotid artery ligation)	ischemia/ reperfusion	i.p.	25, 50 and 100 mg/kg/day (2 weeks)	Cell death reduction and ferroptosis inhibition	
**Antioxidant activity, ** **, Attenuated neuronal necrosis and MDA**	In vivo	Rat)(CCH model)	Chronic cerebral hypoperfusion	oral gavage	25, and 50 mg/kg	Spatial learning and memory deficits improvement using MWM and producedneuroprotective effects on neuronal damages	
**Inhibition of COX-2 activity**	In vivo	Rat (LPS)	Epilepsy	i.p.	100 mg/kg	Prevented the proconvulsant effect of lipopolysaccharide	
**Anti-apoptotic activity:** **-Decreased the level of caspase-3** **-Involvement of PI3K/Akt pathway**	In vivo	Mice	focal cerebral ischemia/reperfusion injury	i.p.	50 mg/kg	protective effects in a middle cerebral artery occlusion in mice	
**Anti-inflammatory**	In vivo	Rat (Focal Cerebral Ischemia–Reperfusion Model)	ischemic stroke	i.p.	10, 20, and 40 mg/kg	A potential therapy for cerebral ischemia injury	
**suppressing** **MAPK/JNK-NF-κB signaling pathway**	In vitro	SH-SY5Y cells (Fe2+-induced apoptosis)	Anti-apoptosis	-	64 and 333 μmol/L)	Protected neuroblastoma SH-SY5Y cells against Fe(2+)-induced apoptosis	
**Anti-inflammatory Antioxidant,** **Anti-apoptosis**	In vivo	Rat (LPS)	Major depressive disorder	i.p.(5 days)	20 mg/kg	Ameliorated LPS-induced neurodegeneration and depressive-like behaviors in rats.	
**TRPM7 inhibitor,** **Reduced intracellular zinc translocation**	In vivo	Rat (global cerebral ischemia model)	Cerebral Ischemia	i.p. (3 days)	50 mg/kg	havingtherapeutic potential after global cerebral ischemia	
**TRPM7 inhibitor,** **Antioxidant,** **Reduced intracellular zinc accumulation**	In vivo	Rat (lithium-pilocarpine induced seizure)	seizure	i.p.(3 days)	50 mg/kg	Having high therapeutic potential for reducing neuronal death caused by seizure	
**Antioxidant** **Attenuated DNA damage**	In vivo	Rat (propiconazole)	anxiety, Impairment of cognitive memory	Oral gavage (60 days)	50 mg/kg	ameliorated behavioral disturbances and DNA damage	
**Inhibited TRPM7, Increased Bcl2/Bax and p-Akt/t-Akt protein ratios, reduced cleaved caspase-3 protein expression**	In vitro; In vivo	HEK293 cells over-expression of TRPM7 and TRPM7-like current in hippocampal neurons	Neonatal ischemic injury	i.p.	*In vitro*: >200 μM *In vivo*:30 and 50 mg/kg	Enhanced behavioral function and decreased brain infarct volume and inhibiting pro-apoptotic signaling	

Bax, Bcl2-associated X protein; Bcl2, B-cell lymphoma 2; CCH, chronic cerebral hypoperfusion; COX-2, cyclooxygenase-2; GPx4, glutathione peroxidase; i.p., Intraperitoneal; LPS, lipopolysaccharide; MAPK/JNK-NF-κB , mitogen-activated protein kinases/ c-jun N-terminal- nuclear factor-κB; MDA, malondialdehyde; p-Akt/t-Akt, phosphorylated protein kinase B/ total protein kinase B; PI3K/Akt, phosphatidylinositol-3-kinase/protein kinase B; TRPM7, transient receptor potential melastatin

## Conflicts of interest

The authors have declared that there is no conflict of interest.
